# Ionic transport in hybrid lead iodide perovskite solar cells

**DOI:** 10.1038/ncomms8497

**Published:** 2015-06-24

**Authors:** Christopher Eames, Jarvist M. Frost, Piers R. F. Barnes, Brian C. O'Regan, Aron Walsh, M. Saiful Islam

**Affiliations:** 1 Department of Chemistry, University of Bath, Bath BA2 7AY, UK; 2 Department of Physics, Imperial College London, London SW7 2AZ, UK

## Abstract

Solar cells based on organic–inorganic halide perovskites have recently shown rapidly rising power conversion efficiencies, but exhibit unusual behaviour such as current–voltage hysteresis and a low-frequency giant dielectric response. Ionic transport has been suggested to be an important factor contributing to these effects; however, the chemical origin of this transport and the mobile species are unclear. Here, the activation energies for ionic migration in methylammonium lead iodide (CH_3_NH_3_PbI_3_) are derived from first principles, and are compared with kinetic data extracted from the current–voltage response of a perovskite-based solar cell. We identify the microscopic transport mechanisms, and find facile vacancy-assisted migration of iodide ions with an activation energy of 0.6 eV, in good agreement with the kinetic measurements. The results of this combined computational and experimental study suggest that hybrid halide perovskites are mixed ionic–electronic conductors, a finding that has major implications for solar cell device architectures.

## Abstract (web summary)

Understanding the mechanism of ionic transport in organic–inorganic halide perovskites is crucial for the design of future solar cells. Here, Eames *et al.* undertake a combined experimental and computational study to elucidate the ion conducting species and help rationalize the unusual behaviour observed in these perovskite-based devices.

## Graphical abstract

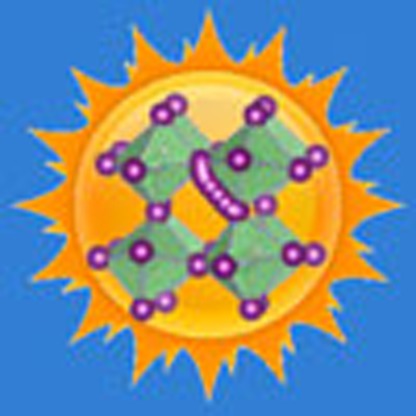

Solar cell materials based on organo-lead halide perovskites are attracting extraordinary attention on account of the rapid rise in their solar-to-electricity conversion efficiencies^1^,^2^,^3^,^4^,^5^. Other key features of these hybrid perovskites include their ease of solution-based processing at low temperature, and their strong optical absorption, although there are significant stability issues. Devices based on methylammonium lead iodide, CH_3_NH_3_PbI_3_, have dominated most research^6^,^7^,^8^,^9^,^10^,^11^,^12^,^13^,^14^,^15^,^16^,^17^,^18^,^19^,^20^,^21^,^22^,^23^,^24^,^25^,^26^,^27^,^28^,^29^,^30^,^31^,^32^, as so far they exhibit the highest power conversion efficiencies (>20%); these values are comparable to those of the best thin-film solar cells based on Cu(In,Ga)Se_2_ or CdTe, but greater than those of conventional dye-sensitized or organic solar cells. The ABX_3_ perovskite-type structure, illustrated in Fig. 1, is comprised of an extended framework of corner-sharing PbI_6_ octahedra with the methylammonium cation (CH_3_NH_3_^+^) occupying the central *A* site and surrounded by 12 nearest-neighbour iodide ions.

Inorganic perovskite oxides have been intensively researched for decades in connection with a rich variety of solid-state magnetic, ferroelectric and conduction properties. In particular, it is well known that numerous perovskite oxides (for example, Sr- and Mg-doped LaGaO_3_) exhibit high ionic conductivity mediated by defect species (typically oxide-ion vacancies), making them useful in applications such as solid oxide fuel cells^33^,^34^. In addition, ionic conductivity studies of inorganic perovskite halides (for example, CuPbI_3_ and CsPbCl_3_) have reported low activation energies for the migration of halide-ion vacancies^35^,^36^,^37^.

Diffusion of intrinsic ionic defects in organo-lead halide perovskites has important implications in terms of the long-term stability and performance of perovskite solar cell devices. In these hybrid perovskites, however, the exact nature of the mobile ionic species and migration activation energies are poorly understood.

Charge transport and resistivity studies of hybrid halide perovskites have largely focussed on their semiconducting (electron/hole transport) behaviour, and indicate high carrier mobility and long minority-carrier diffusion lengths^10^,^11^,^12^. Current–voltage characteristics show a hysteresis in photovoltaic performance^13^,^14^,^15^,^16^,^17^; this has been speculated to be related to ion migration^17^,^18^. Xing *et al.*^22^ suggest that the non-radiative pathways involved in electron–hole recombination may include bulk defects such as vacancies. From spectroscopic impedance studies, Dualeh *et al.*^31^ suggest that the hybrid perovskites might exhibit ionic charge transport in addition to electronic conduction. More recently, Xiao *et al.*^32^ ascribe the field-switchable photovoltaic effect to the drift of charged ions or vacancies, although they provided no direct evidence for ion diffusion. In addition to ion migration, ferroelectric and charge-trapping effects have also been suggested as possible mechanisms^13^. Recent analysis of quasielastic neutron scattering data^23^, as well as polarization and piezoresponse force microscopy measurements^38^ suggests that ferroelectric behaviour in CH_3_NH_3_PbI_3_ is unlikely to be the dominant mechanism behind the observed hysteresis. Simple capture and release of photogenerated charges in bulk or interfacial defects is also unlikely due to the magnitude and duration of the changes in photocurrent during relaxation following biasing.

Previous electronic structure calculations have been used to examine band structures, spin–orbit coupling and intrinsic defects in CH_3_NH_3_PbI_3_ (refs 39, 40, 41, 42, 43, 44, 45, 46, 47, 48, 49, 50, 51). The defect studies^45^,^46^ suggest that Pb^2+^, I^−^ and CH_3_NH_3_^+^ (MA^+^) vacancies create shallow donor or acceptor levels. Recent simulation work^41^ shows the prevalence of ionic over electronic disorder in CH_3_NH_3_PbI_3_ with low formation energies for Schottky-type defects associated with anion and cation vacancies, shown in the following reaction in the Kröger–Vink notation for the full Schottky defect,


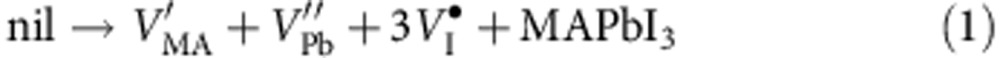


where nil represents the perfect CH_3_NH_3_PbI_3_ lattice, *V* indicates a vacancy, subscripts the ionic species and superscripts the effective defect charge (a dot for each positive charge and prime for each negative charge); recent results^41^ suggest a significant equilibrium concentration of I^−^, Pb^2+^ and CH_3_NH_3_^+^ vacancies at room temperature, which could support vacancy-mediated diffusion.

Macroscopic conductivity or diffusion experiments, however, have so far not enabled the mobile defect species or the atomistic transport mechanism to be identified. Here, we investigate key issues related to intrinsic defect migration in CH_3_NH_3_PbI_3_ using first-principles techniques combined with kinetic experiments monitoring the photocurrent relaxation of devices, extending our previous work on hybrid perovskites^39^,^40^,^41^ and ion transport in perovskite-type oxides^33^,^52^. We have carried out a detailed examination of the pathways and relative activation energies for the vacancy-mediated migration of I^−^, Pb^2+^ and CH_3_NH_3_^+^ ions, and compared these values directly with kinetic data extracted from a working CH_3_NH_3_PbI_3_ solar cell. The results support a mechanism of vacancy-mediated ion diffusion, pointing to mixed ionic–electronic conduction in these hybrid perovskites. The implications for perovskite solar cell operation and stability are discussed.

## Results

### Migration mechanisms and energies

It is well established that the mobile ionic species in the solid state is associated with some type of vacancy or interstitial defect, the concentration of which is controlled by intrinsic Schottky and Frenkel defect reactions, non-stoichiometry or aliovalent doping^33^,^34^,^53^. In materials with the ABX_3_ perovskite structure, vacancy-mediated diffusion is the most common process, which is further supported for CH_3_NH_3_PbI_3_ by the ease of formation of Schottky disorder (reaction (1)), with an intrinsic concentration of I^−^, Pb^2+^ and CH_3_NH_3_^+^ vacancies predicted to exceed 0.4% at room temperature^41^. Interstitial migration has not been observed in inorganic perovskite oxides or halides due to the lack of interstitial space in such close-packed structures.

**Figure 2:**
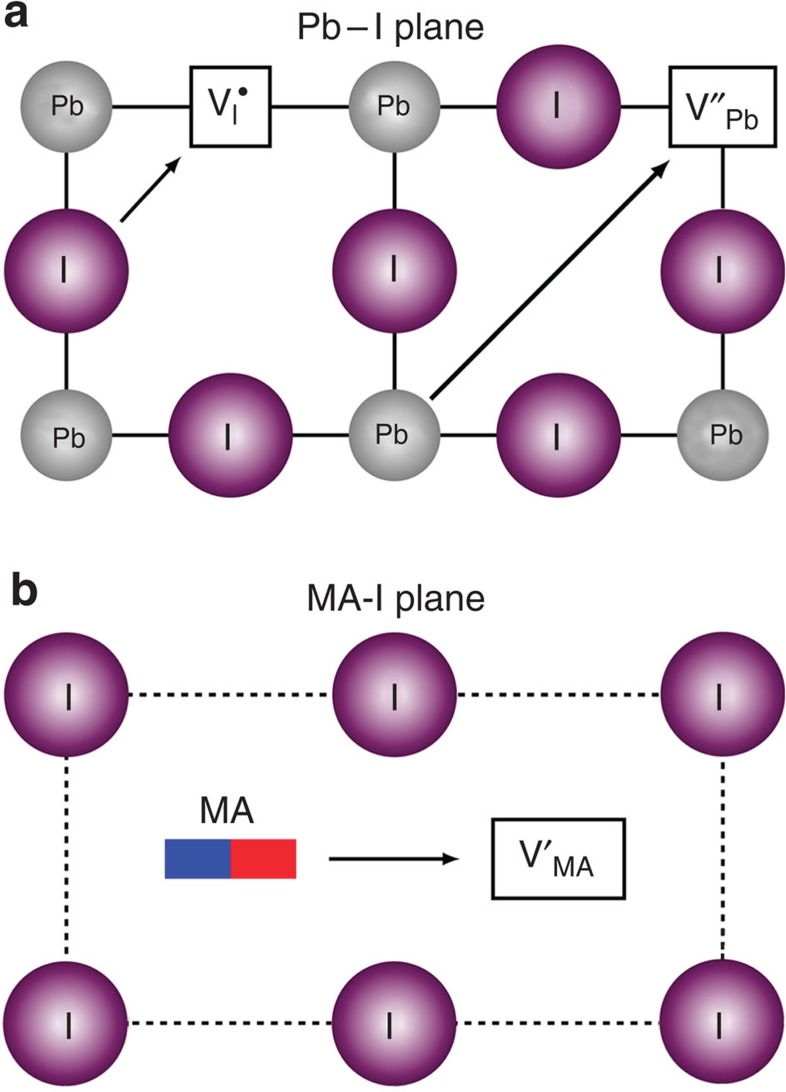
Transport mechanisms in the CH_3_NH_3_PbI_3_ perovskite structure. Schematic illustration of the three ionic transport mechanisms involving conventional vacancy hopping between neighbouring positions: (**a**) I^−^ migration along an octahedron edge; Pb^2+^ migration along the diagonal direction <110>; (**b**) CH_3_NH_3_^+^ migration into a neighbouring vacant *A*-site cage involving motion normal to the unit cell face composed of four iodide ions.

In this study, three vacancy transport mechanisms involving conventional hopping between neighbouring positions (illustrated in Fig. 2) were considered. These were (i) I^−^ migration along an octahedron edge; (ii) Pb^2+^ migration along the diagonal (<110> directions) of the cubic unit cell; (iii) CH_3_NH_3_^+^ migration into a neighbouring vacant *A*-site cage. Energy profiles for these mechanisms were mapped out by performing a series of transition-state calculations between adjacent equivalent sites. Particular care has to be taken to ensure the starting and end point configurations are well converged to avoid anomalously low migration energies. This approach allows the energy barrier to ion migration to be determined, and has been used successfully in previous studies on oxide-ion, proton and metal-ion migration in perovskite oxides^52^,^54^. One particular challenge is to adequately describe the polarization response to the change in position of the migrating ion, which involves a combination of electronic polarization and lattice relaxation; this required the large supercells employed here to minimize any spurious interactions.

The migration activation energies for the three mechanisms are reported in Table 1. The lowest activation energy of 0.58 eV suggests favourable vacancy-assisted diffusion of iodide ions. A higher activation energy of 0.84 eV is found for CH_3_NH_3_^+^ migration, which involves motion through the unit cell face or bottleneck comprising four I^−^ ions (ion radius of 2.20 Å); this value is probably at the lower limit since we have considered the ideal migration path normal to the unit cell face. In addition, neutron diffraction^55^ and quasielastic neutron scattering^23^ studies confirm a high level of orientational motion of the CH_3_NH_3_^+^ ion at room temperature, which is likely to further inhibit long-range transport. Table 1 shows a high energy barrier for Pb^2+^ vacancy migration, suggesting an immobile Pb sublattice. This result is consistent with the thermodynamic stability of the inorganic lead iodide sublattice of the perovskite structure. The high activation energy for Pb^2+^ transport also suggests that such cation diffusion is likely to be rate controlling for crystal growth processes.

From the calculated activation energies a diffusion coefficient can be estimated. Assuming a Boltzmann-like barrier-hopping process with a typical attempt frequency of 10^12^ Hz for ionic species at a temperature of 320 K, we estimate a diffusion coefficient of 10^−12^ cm^2^ s^−1^ for I^−^ ions, which is four orders of magnitude higher than the value of 10^−16^ cm^2^ s^−1^ for CH_3_NH_3_^+^, suggesting negligible diffusion of CH_3_NH_3_^+^ ions. These results clearly indicate that hybrid halide perovskites are mixed ionic–electronic conductors with iodide ions as the majority ionic carriers. A key factor for ionic conductivity will be the level of intrinsic iodide ion vacancies in the material, which will be sensitive to the synthesis conditions (equilibrium or non-equilibrium) and thermal processing routes. It is recognized that, as in binary lead halides, there are stability and photodecomposition issues with these hybrid lead perovskites^1^,^2^,^56^, which require further investigation.

**Figure 3:**
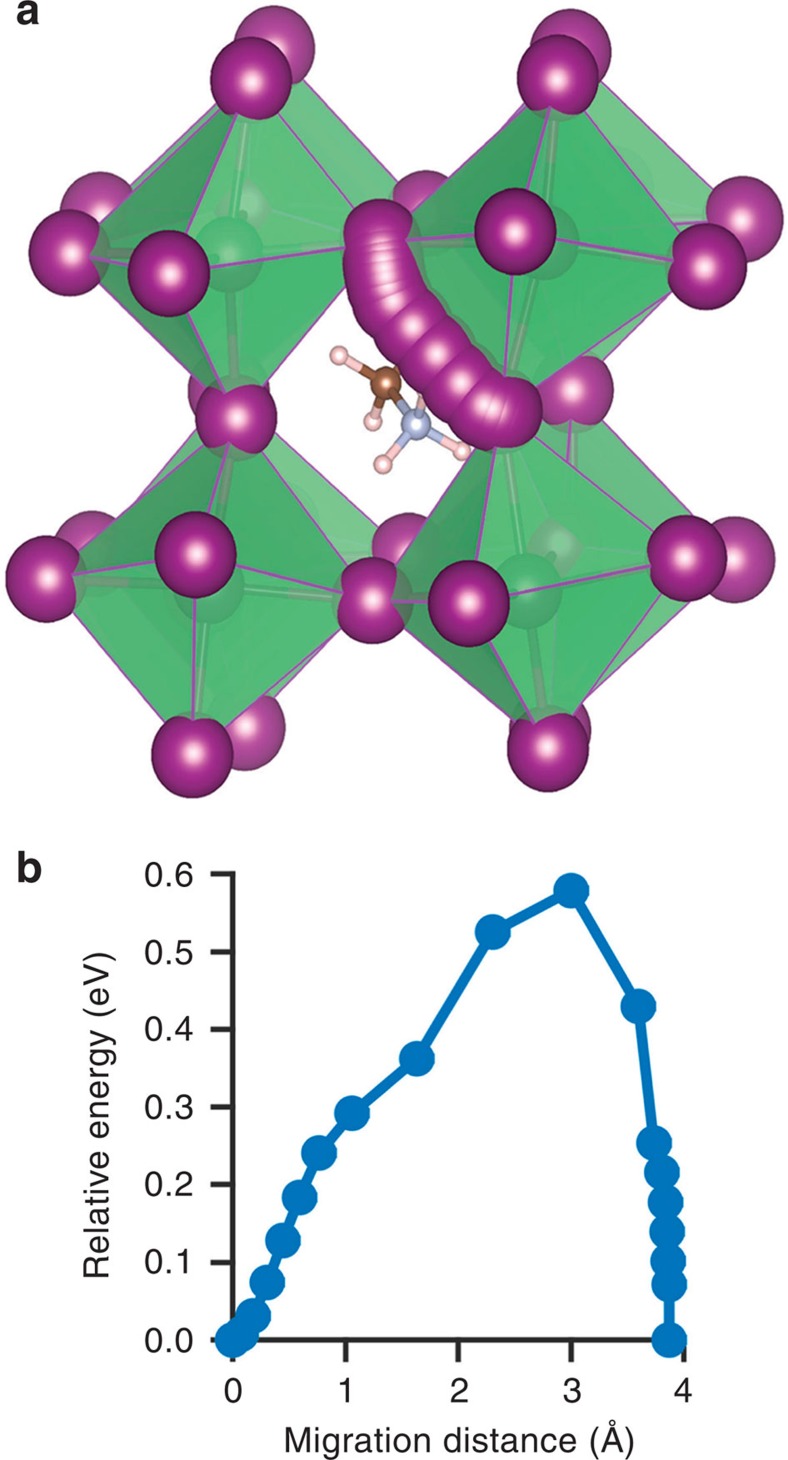
Iodide ion vacancy migration from density functional theory calculations. (**a**) Calculated migration path indicating a slightly curved path and local relaxation/tilting of the octahedra. (**b**) Corresponding energy profile.

With regard to mechanistic features, it is often assumed that the migrating ion takes the shortest path between adjacent sites, that is, a direct linear jump. However, detailed analysis of the migration paths for the iodide ion vacancy mechanism reveals a small deviation from the linear route involving a curved path between iodine sites, with the saddle point slightly bowed away from the neighbouring Pb ion (shown in Fig. 3 together with the corresponding energy profile); such an atomic-scale mechanism is difficult to extract from an experiment alone. It is worth noting that analogous curved paths have been found from both atomistic simulation^52^ and neutron diffraction studies^33^,^57^ of oxide-ion conduction in inorganic perovskite oxides such as doped LaGaO_3_. Similar mechanisms may feature in related hybrid halide perovskites.

### Kinetic measurements

Several recent studies have probed current–voltage hysteresis in hybrid perovskite solar cells^13^,^14^,^15^,^16^,^17^. However, there is currently an absence of temperature-dependent kinetic data. Hysteresis is frequently observed when performing a photocurrent–voltage scan on the device. If the voltage is held at forward bias and then swept from forward to reverse, the power conversion efficiency inferred from the measurement can be as much as 90% higher than that inferred if the current–voltage scan proceeds from reverse to forward bias. The size of the difference depends on the scan rates, the preconditioning voltage and time and the light level. Recent studies indicate that there is no direct influence of light on the hysteresis in the photocurrent^15^,^17^. Light may have an indirect effect on hysteresis by generating a photovoltage that influences device performance in a similar way to the application of an external bias. The effect of sweeping voltage with time can be decoupled from the hysteresis phenomenon by making stepped chronophotoampereometry measurements where the relaxation of the photocurrent towards a steady-state value at short circuit is observed following application of a constant bias in the dark.

**Figure 4:**
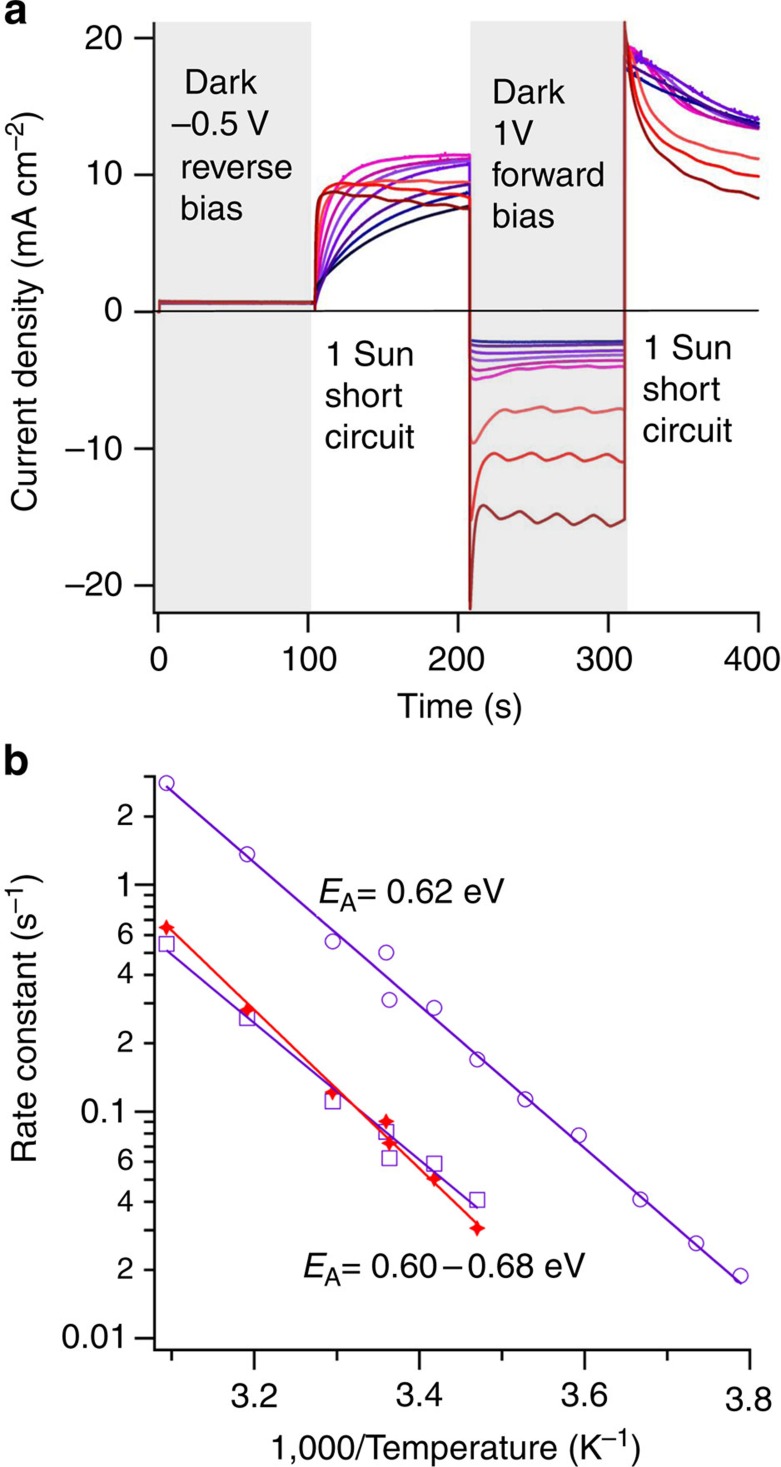
Chronophotoamperometry measurements of a perovskite-based cell. (**a**) The measurement sequence in a d-TiO_2_/CH_3_NH_3_PbI_3_/spiro-OMeTAD/Au cell is indicated; measured temperatures (to the nearest 0.5 °C) of the devices were −9.5 (dark blue), −5.5, 0.5, 5, 10.5, 15, 19.5, 24.5, 30, 40 and 50 °C (dark red). The dark current under forward bias was very sensitive to fluctuations in the controlled temperature; no time constants were taken from the dark current. (**b**) Arrhenius plot of the rates of photocurrent relaxation. Fits (purple lines) to the fast (*k*_1_) and slow (*k*_2_) components of bi-exponential to the photocurrent rise at 1 sun equivalent light intensity following reverse bias at −0.5 V in the dark (open circles and squares, respectively). The activation energy of the fast component evaluated between −9.5 and 50 °C was *E*_A_=0.62 eV. The activation energy for the slow component evaluated between 15 and 50 °C was *E*_A_=0.60 eV (the measurement duration was insufficient to reliably estimate the slow component at lower temperatures). The photocurrent rise at 50 °C did not reach a stable plateau and started to decline after its peak; we did not fit to this portion of the curve. The red crosses show the rates inferred from a single exponential fit to the tail of the photocurrent decay (*k*_3_) following forward bias at 1 V in the dark; the corresponding activation energy is 0.68 eV (red line) evaluated between 15 and 50 °C. Given the spread of points, we consider that the range of activation energies determined is similar to the uncertainty of the estimation.

By measuring the temperature- and time-dependent photocurrent following forward and reverse biasing in the dark, the rate at which the cell relaxes to equilibrium can be determined and thus activation energies for the relaxation of the device can be estimated (see Fig. 4a,b). The temperature range covered in this study is representative of typical device-operating temperatures but below the second-order tetragonal-to-cubic phase transition. Following dark reverse bias conditions, the photocurrent rise at short circuit was fitted well by a bi-exponential. The time constant for the initial part of the photocurrent rise after reverse biasing varied between 53 s at −9.5 °C and 0.36 s at 50 °C. The time constant for the slower phase of the rise was six times longer, with values very similar to those obtained by fitting to single exponential functions to the tail of the photocurrent decay following forward biasing in the dark (Fig. 4a). The shape of the photocurrent relaxations remained relatively constant but scaled with time according to the temperature, implying that only the kinetics of the relaxation process had changed. The activation energies for short-circuit photocurrent relaxation from both forward (1 V) and reverse bias (−0.5 V) preconditioning were similar: 0.68 eV and 0.60–0.62 eV, respectively. This similarity, combined with the observation that the slow phases of both the rise and fall of the photocurrent have approximately the same time constants for a given temperature, suggests that the underlying process controlling the relaxation is reversible.

The measured activation energies for hysteresis, 0.60–0.68 eV, are very close to the value calculated for the migration of iodide ion vacancies, 0.58 eV. Our measured and calculated values are also compatible with experimental ionic conductivity studies of inorganic perovskites, which have reported similar activation energies for halide-ion vacancy conduction in perovskite halides (for example, CsPbCl_3_)^35^,^36^,^37^ and oxide-ion vacancy transport in perovskite oxides^33^,^34^. In contrast, the activation energies calculated for the migration of MA^+^ and Pb^2+^ vacancies (0.84 and 2.31 eV, respectively) are higher than the measured values, confirming that significant migration of these ions is unlikely at the temperatures and timescales examined.

The diffusion length of an iodide vacancy on the timescale of a photocurrent relaxation at room temperature (around 10 s) using our estimated diffusion coefficient (10^−12^ cm^2^ s^−1^) is of the order of 30 nm. The corresponding drift distance in this time due to an electric field that might be found in a device of this thickness (1 V dropped uniformly across 500 nm) is around 80 nm. As an example, the change in potential across a layer of accumulated vacancies of thickness *w* of 10 nm yields a value of ≈0.6 V, using the relation *qw*^2^*N*/(2*ɛ*_0_*ɛ*_r_) assuming a relative permittivity (*ɛ*_r_) of 24 and 0.4% Schottky defects (*N*≈1.6 × 10^19^ cm^−3^) at room temperature^41^. Hence, a change of this magnitude in electric potential in the perovskite adjacent to electrodes would certainly result in a significant variation in the observed photocurrent in a solar cell.

**Figure 5:**
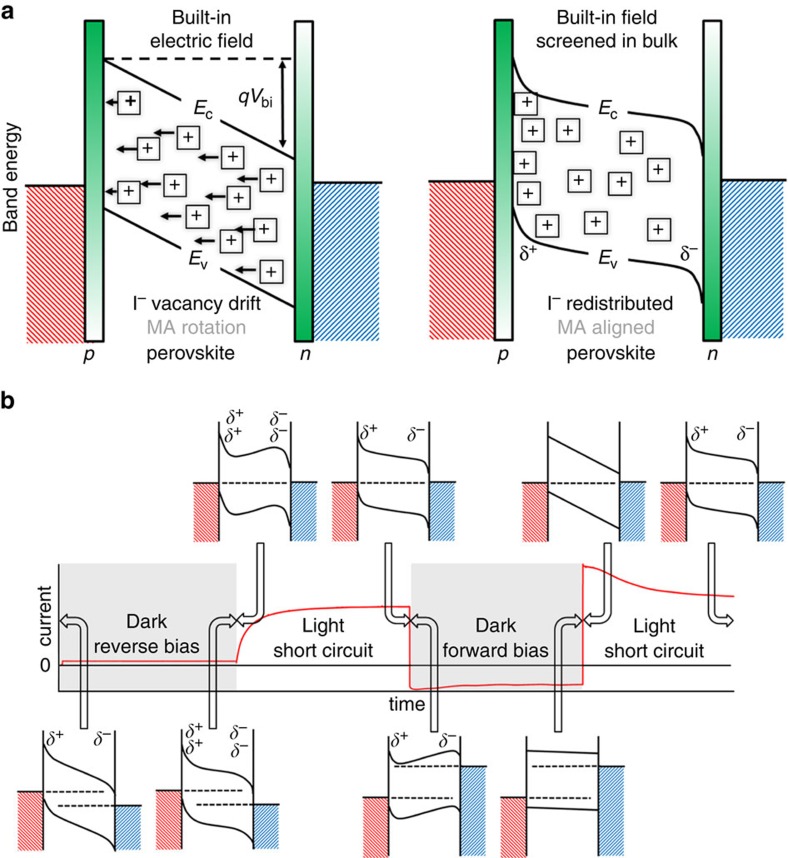
Influence of iodide ion vacancies on band energies of a perovskite thin film. (**a**) Schematic diagrams indicating the influence of vacancy drift on the band energies of a p-i-n device at short circuit. *E*_C_ is the conduction band energy, *E*_V_ is the valence band energy and *V*_bi_ is the built-in potential. Iodide ion vacancies are represented by the squares with ‘plus' signs. Implicit in the diagram is that the vacancies with effective positive charges are balanced by immobile cation vacancies (not shown) with effective negative charges. (**b**) Hypothesized energy level configurations corresponding to different bias conditions and times during the chronophotoamperometry measurements. The variation in the conduction and valence bands corresponds to the redistribution of iodide ion vacancies to and from interfaces with different applied potentials and times.

Migration of iodide ion vacancies under the influence of an electric field could change the photogenerated charge collection efficiency of devices with time and so helps to explain hysteresis. The possible influence of iodide ion vacancies on band energies of a perovskite thin film device and its interfaces is described in Figure 5. We suggest the following model to explain the chronophotoamperometry measurements (see Fig. 5b). When the cell is short-circuited, a built-in electric field is present in the perovskite layer due to the difference in the work functions of the contacts. This could result in the migration of iodide ion vacancies towards the contacts (while immobile vacancies remain fixed), partially screening the field. As discussed above, we expect the magnitude of the short-circuit photocurrent to be controlled by the extent to which the electric field is screened (as also recently suggested by Tress *et al.*^17^). A reduced internal field within the device (due to ionic screening) would lead to less efficient collection of photogenerated charge carriers. Prolonged poling of the device under forward bias reduces (or reverses) the built-in field, which could allow a dissipation of ionic charge from the contacts by diffusion. This tempering would result in more efficient collection of photogenerated charges when the device is returned to short circuit, since the built-in field is no longer screened by accumulated ionic or vacancy charge. Conversely, holding the device under reverse bias would accentuate the migration of ions and vacancies to the interfaces, resulting in less efficient collection of photogenerated charges when the device is returned to short circuit.

Within this model, the rate at which the short-circuit photocurrent relaxes towards equilibrium following poling, under either forward or reverse bias, gives an indication of the rate of ion diffusion. The temperature dependence of iodide ion migration would then correspond to the measured activation energy. The agreement between the measured and predicted activation energies supports ionic transport as one of the primary causes of the anomalous hysteresis in current–voltage curves^13^,^14^,^15^,^16^,^17^, and the giant switchable photovoltaic effect^32^.

The detailed behaviour may be more complex owing to additional contributions from, for example, electronic charge redistribution (at faster timescales). Although this investigation is not exhaustive in terms of possible polarization phenomena and direct observation of ion migration, it does highlight a crucial area for further work on this fascinating system. Indeed, large-scale molecular dynamics simulations could be used to explore diffusion mechanisms and defect association effects, while isotope diffusion measurements would provide a direct probe for determining the extent of ion transport in these materials.

## Discussion

Our combined computational and experimental study has provided new atomic-scale insights into the ion transport mechanisms operating in the hybrid perovskite CH_3_NH_3_PbI_3_. We find facile vacancy-assisted diffusion of iodide ions, with good agreement with activation energies derived from kinetic measurements on a CH_3_NH_3_PbI_3_-based solar cell. A key factor influencing the conduction of iodide ions (as the majority ionic carriers) will be the levels of intrinsic iodide ion vacancies in the material, which will be sensitive to the fabrication conditions and sample thermal history.

Our study provides a framework for understanding ionic transport phenomena in organic–inorganic halide perovskites, including the influence of the migration of iodide ion vacancies to and from interfaces in solar cell devices. Such ion migration has been suggested as a factor contributing to their unusual behaviour, including current–voltage hysteresis and a giant dielectric response at low frequencies. The picture emerging is that hybrid halide perovskites are mixed ionic–electronic conductors. This behaviour has major repercussions for interpreting solar cell device performance and degradation pathways, and for the design of future architectures.

## Methods

### Computational

The theoretical framework for modelling ion transport in the solid state is well developed^53^, and has been extensively validated for perovskite oxides^52^,^54^. The CH_3_NH_3_PbI_3_ perovskite structure and energies were calculated using density functional theory methods (employing the *ab initio* code VASP^58^). A 4 × 4 × 4 supercell (768 atoms) of the pseudo-cubic unit cell was modelled; a plane wave cutoff energy of 500 eV, *k*-point sampling at the gamma point, PAW pseudopotentials^59^ and the PBEsol exchange-correlation functional were employed. For structure relaxation, forces were converged to <0.01 eV Å^−1^. For the hybrid perovskites, PBEsol reproduces the experimental crystal structure in good agreement with neutron diffraction data, and also describes the finite temperature behaviour (lattice vibrations and dynamics) in agreement with Raman and neutron scattering. We obtained an average lattice parameter of 6.28 Å for CH_3_NH_3_PbI_3_, in good agreement with the observed values of 6.28–6.32 Å from X-ray and neutron diffraction experiments (Table 2)^11^,^55^,^60^,^61^. The defect results using PBEsol are very similar to those using other functionals, for example, see discussions of LDA/PBE in ref. 50. For migration barriers, the expanded lattice volume for standard GGA functionals (such as PBE) could be a major source of error, which PBEsol avoids.

Activation energies for diffusion processes can be computed from the total energy difference between the diffusing species in their ground-state configuration and at the saddle point of the diffusion process. Migration mediated by ion vacancies was examined using nudged elastic band and constrained energy minimization methods^58^; in the latter, the migrating species is propagated along the migration direction in a series of small steps with all unconstrained degrees of freedom relaxed at each step. While in a cubic lattice with disordered CH_3_NH_3_^+^ ions, each iodine site is equivalent, for an ordered cell that we used there are distinct apical and equatorial positions. We previously confirmed^41^ that the vacancy formation energy is similar on both sites; here, we performed nudged elastic band calculations involving each iodine position, and find that the resulting activation energies are within 0.01 eV, and also insensitive to the alignment of methylammonium ions.

### Solar cell fabrication

Methylammonium lead iodide solar cells were fabricated using a modification of standard recipes^5^. Fluorine-doped tin oxide (F:SnO_2_)-coated glass (TEC 15, Hartford Glass Co. Inc.) was patterned by etching with zinc powder and 2 M HCl, cleaned with deionized H_2_O, isoproponal and heated at 450 °C for 30 min. A dense titanium dioxide (∼80 nm) blocking layer (d-TiO_2_) was deposited on SnO_2_:F glass substrates via spray pyrolysis and heated at 450 °C for 30 min^62^. A solution of 2.64 M methylammonium iodide and 0.88 M lead chloride in dry DMF was spun at 2,000 r.p.m. (acceleration 1,000 r.p.m. s^−1^) onto the substrate and dried at room temperature for 30 min before heating at 90 °C for 3 h in a glove box. Studies suggest that negligible chloride remains in the film following this preparation. The resulting CH_3_NH_3_PbI_3_ perovskite film thickness was ∼500 nm. A solution of 61.6 mM spiro-OMeTAD, 55 mM tBP and 26 mM LiTFSI in dry chlorobenzene was then spun onto the perovskite, as above, to create the hole-transporting layer. A gold electrode was evaporated onto this with an active area of 0.08 cm^2^. The cell was sealed in the glove box using a glass coverslip and a surlyn gasket.

### Kinetic measurements

Chronophotoampereometry measurements were made using the TRACER system^15^,^63^. The current and voltage were measured while bias light (from white light-emitting diodes), applied voltage and short-circuit/open-circuit status were modulated in an arbitrary sequence of steps. The modulation was accomplished with three MOSFET switches possessing <1 μs switching time and synchronicity (for both the optical and electrical steps). The temperature of the cells was controlled to within ±1 °C using a Peltier cooler/heater to which the devices were coupled using thermal paste. Devices were stabilized for 5 min at each temperature before measurement. Illumination by the light-emitting diodes was also found to result in <1 °C temperature increase of the device. The chronophotoampereometry measurement sequence was as follows. The current through the cell was recorded with a reverse bias of −0.5 V for 102 s, the device was then switched to short-circuit mode (0 V) and the light simultaneously turned on (with 1 sun equivalent absorbed photon flux) for a further 102 s. The light was then turned off and a forward bias of 1 V was simultaneously applied to the cell for 102 s. The cell was then returned to short-circuit mode (0 V) and the light simultaneously switched on for a final 102 s.

Empirically, the rise in the photocurrent (*J*_sc_) towards equilibrium (*J*_sc,equilibrium_) after reverse bias was found to be well fit by a double exponential function:


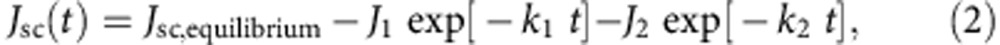


where *t* is the time after switching, *k*_1_ and *k*_2_ are the rate constants (units: s^−1^) for the fast and slow phases of the rise and *J*_1_ and *J*_2_ are constants. The decay of the photocurrent towards equilibrium was observed to have a more complicated functional form with a shoulder in the decay at early times. The tail of this decay could be well fit by a single exponential function:


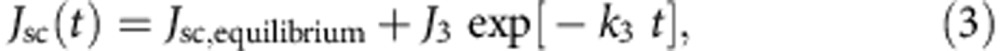


where *k*_3_ is the rate constant for the decay and *J*_3_ is a constant. As discussed in the main text, we hypothesize that these phenomenological rates of photocurrent relaxation (*k*_1_, *k*_2_ and *k*_3_) are related to the formation or removal of energetic barriers in the material, which change the efficiency of photogenerated charge carrier collection^17^.

The barriers result from the motion of ionic charge to or from the interfaces following a change in the electric field across the device caused by switching the bias voltage state. Measurements were taken as a function of temperature (*T*) from 263 to 323 K, representative of device-operating temperatures but below the tetragonal-to-cubic phase transition. Associated activation energies (*E*_A_) were determined by fitting the expression ln[*k*]=*C*—*E*_A_/(*k*_*B*_ *T*) to an Arrhenius plot of the rate (*k*) data, where *C* is a constant and *k*_B_ is Boltzmann's constant. Although the activation energies are derived from the rates of photocurrent relaxation, they are likely to be related to the underlying mechanism that causes the change in photocurrent.

## Additional information

**How to cite this article:** Eames, C. *et al.* Ionic transport in hybrid lead iodide perovskite solar cells. *Nat. Commun.* 6:7497 doi: 10.1038/ncomms8497 (2015).

## Figures and Tables

**Figure 1:**
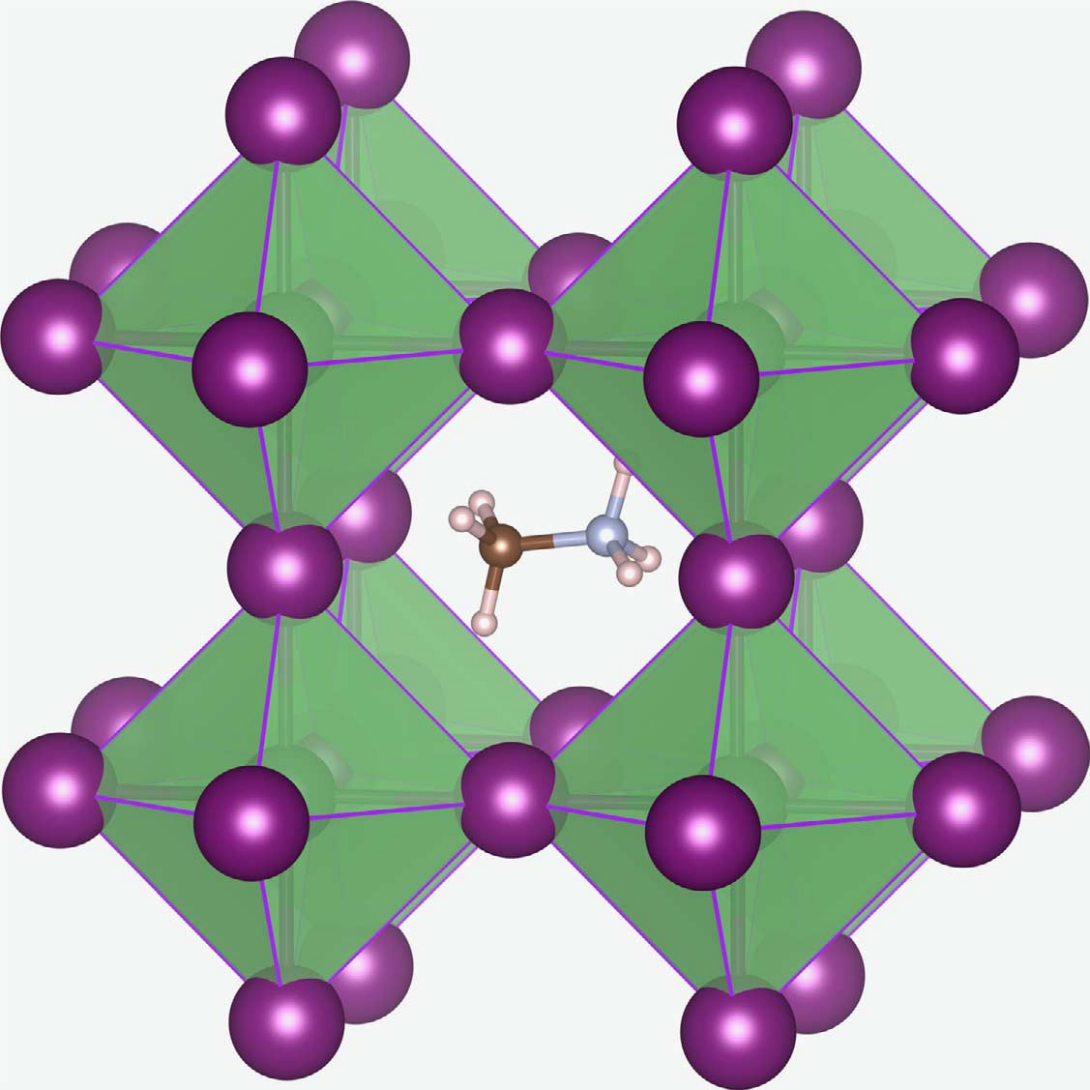
Perovskite structure of CH_3_NH_3_PbI_3_. Methylammonium cation (CH_3_NH_3_^+^) occupies the central *A* site surrounded by 12 nearest-neighbour iodide ions in corner-sharing PbI_6_ octahedra.

**Table 1:** Calculated activation energies for ionic migration in CH_3_NH_3_PbI_3_.

**Migrating vacancy**	**Defect notation**	***E***_**A**_ **(eV)**
I^−^	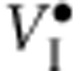	0.58
Pb^2+^	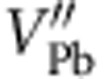	2.31
CH_3_NH_3_^+^ (MA^+^)	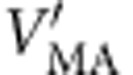	0.84

The migration of ion species is mediated by vacancy defects.

**Table 2:** Structural parameters of CH_3_NH_3_PbI_3_.

**Structural parameter (Å)**	**DFT (this study)**	**X-ray**^60^ **diffraction**	**X-ray**^11^ **diffraction**	**Neutron**^55^ **diffraction**	**X-ray**^61^ **diffraction**
Cubic lattice parameter	6.28	6.28	6.31	6.32	6.29
I–I separation	4.45	—	4.46	4.47	—
Pb–I bond length	3.16	—	3.16	3.16	—
C–N bond length	1.48	—	1.48	1.35*	—
C–H bond length	1.10	—	—	0.99*	—
N–H bond length	1.04	—	—	0.99*	—

DFT, density functional theory.

Calculated (DFT) parameters are compared with experimental data for the cubic phase.

^*^Due to the manner in which orientational disorder is fitted to neutron diffraction data for the cubic system, these bond lengths from ref. 55 represent an underestimate; in the refinement of the orthorhombic structure they used bond lengths with soft constraints of 1.46 Å (C–N), 1.13 Å (C–H) and 1.00 Å (N–H), which are comparable to our calculated values.
